# TRPV4‐Mediated Mechanosensing Regulates the Endothelial‐to‐Mesenchymal Transition: Implications for Atherosclerosis

**DOI:** 10.1096/fj.202403198R

**Published:** 2025-12-13

**Authors:** Pritha Mukherjee, Karunakaran R. Sankaran, Mohammad Imran Khan, Suneha G. Rahaman, Shaik O. Rahaman

**Affiliations:** ^1^ Department of Nutrition and Food Science University of Maryland College Park Maryland USA

**Keywords:** aortic endothelial cells, mechanosensing, traction force microscopy, transient receptor potential vanilloid 4

## Abstract

Endothelial‐to‐mesenchymal transition (EndMT) is a crucial biological process where endothelial cells lose their specialized phenotype and acquire mesenchymal characteristics, such as enhanced motility and the ability to produce extracellular matrix components. This process serves dual roles in cardiovascular health and disease. In this study, we investigated the role of mechanosensitive Ca^2+^‐permeant transient receptor potential vanilloid 4 (TRPV4) channels in matrix stiffness‐ and transforming growth factor β1 (TGFβ1)‐induced EndMT. Using primary mouse wild‐type aortic endothelial cells (aECs) and TRPV4‐null aECs, we validated TRPV4 functionality through live Ca^2+^ influx detection in response to selective agonists and antagonists. Employing physiologically relevant hydrogels with varying stiffness that mimic healthy and diseased aortic tissue, we observed that genetic deletion of TRPV4 or its pharmacological inhibition suppressed intracellular matrix stiffening and cellular force generation as quantified by atomic force microscopy analysis and traction force microscopy analysis, respectively, and prevented matrix stiffness‐ and TGFβ1‐induced EndMT. Further analysis revealed that the N‐terminal residues 100–130 of TRPV4 are critical for intracellular matrix stiffening, traction force generation, MLC2 phosphorylation, and EndMT in aECs. Additionally, our findings demonstrated that TRPV4 regulates matrix stiffness‐induced MLC2 activity, thereby modulating EndMT and cellular force generation, identifying a potential mechanism by which TRPV4 activity regulates EndMT in aEC. These results uncover a novel role for TRPV4‐mediated mechanotransduction in regulating EndMT and suggest that TRPV4 could be a promising therapeutic target for addressing cardiovascular diseases.

## Graphical abstract

TRPV4‐dependent mechanotransduction integrates matrix stiffness and soluble biochemical signals to induce endothelial‐to‐mesenchymal transition via the Smad3/MLC2/AKT pathway.

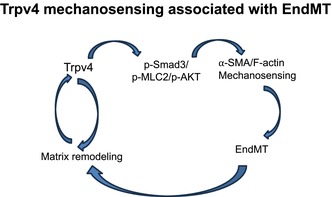

## 1 Introduction

EndMT is a critical biological process in which endothelial cells lose their characteristic phenotype and gain mesenchymal properties, including increased motility and the ability to produce extracellular matrix (ECM) components [1, 2, 3, 4, 5]. This phenomenon plays dual roles in cardiovascular health and disease, acting as a physiological mechanism during development and a pathological driver in adult diseases [1, 2, 3, 4, 5, 6, 7, 8, 9, 10]. EndMT contributes to the structural complexity of the heart by enabling endothelial cells to differentiate into mesenchymal cells, which are integral to the heart's development [6, 8]. Dysregulation of EndMT during this stage can lead to congenital heart defects, highlighting its critical role in orchestrating cardiac development [6, 7, 8]. In the context of cardiovascular diseases (CVDs), EndMT is increasingly recognized as a pivotal contributor to pathological remodeling [1, 2, 3, 4, 5, 6, 7, 8, 9, 10]. It is implicated in conditions such as fibrosis, atherosclerosis, and pulmonary arterial hypertension [1, 2, 3, 4, 5, 6, 7, 8, 9, 10]. For instance, in cardiac fibrosis, ECs undergoing EndMT contribute to the expansion of myofibroblasts, which produce excessive ECM and impair cardiac function [5]. Similarly, in atherosclerosis, EndMT has been linked to the formation of fibrous plaques, which compromise vascular integrity and increase the risk of thrombosis [1, 2, 3].

Molecular signaling pathways such as TGFβ1, Wnt/β‐catenin, and Notch are central regulators of EndMT, and their dysregulation is a hallmark of its pathological activation [1, 2, 3, 4, 5, 6, 7, 8, 9, 10, 11, 12]. Understanding these pathways has spurred interest in therapeutic strategies aimed at modulating EndMT. For example, inhibiting TGFβ1 signaling has shown promise in preclinical models to attenuate fibrosis [13, 14]. Matrix stiffening, a hallmark of many pathological conditions, plays a pivotal role in regulating EndMT [15, 16, 17, 18, 19, 20]. The stiffness of the ECM directly influences cellular behavior by altering mechanical signals that ECs perceive and transduce into biochemical responses, a process known as mechanotransduction. Matrix stiffening synergizes with biochemical signals, particularly the TGFβ1 pathway, a key driver of EndMT [1, 15, 18, 21]. A stiffer matrix facilitates latent TGFβ1 activation from the ECM, increasing its bioavailability and enhancing SMAD2/3 phosphorylation [21, 22, 23, 24]. This promotes the transcription of mesenchymal markers (e.g., α‐SMA, fibronectin) and represses endothelial markers (e.g., VE‐cadherin). Increased ECM stiffness induces cytoskeletal tension in ECs [1, 2, 3, 4, 5]. The resultant remodeling of the actin cytoskeleton generates mechanical stress that further supports the acquisition of a mesenchymal phenotype. This mechanical feedback loop may amplify EndMT in response to a stiffened matrix.

Emerging evidence highlights the role of matrix stiffness in regulating key cellular processes, including gene expression, inflammation, and differentiation [25, 26, 27, 28, 29, 30, 31, 32, 33, 34, 35, 36]. Aortic endothelial cell (aEC) activation and aortic tissue stiffening are increasingly recognized as critical drivers of CVD development and progression [1, 2, 3, 4, 5, 6, 7, 8, 9, 10, 15, 18, 21]. Previous studies, including work from our laboratory, have demonstrated that matrix stiffness influences the activities of various cell types, such as fibroblasts, epithelial cells, and macrophages, affecting migration, differentiation, and myofibroblast activation [25, 26, 27, 28, 29, 30, 31, 32, 33, 34, 35, 36]. These findings suggest the existence of a cellular stiffness sensor in aECs that mediates the link between matrix stiffness and CVD pathology. Mechanosensitive transient receptor potential vanilloid 4 (TRPV4) channels are strong candidates for this role. Published studies, including our own, have shown that TRPV4 channels regulate fibrosis and cell differentiation in various organs in vivo and control fibroblast, macrophage, and dermal epithelial cell function, supporting their potential role as stiffness sensors in CVDs [12, 28, 29, 30, 31, 32, 34, 35, 36, 37]. However, the specific identity of the stiffness sensor in aECs and the associated stiffness‐induced fibrotic signaling pathways remain to be fully elucidated.

TRPV4 is expressed in multiple cell types, including macrophages, epithelial cells, and fibroblasts, and responds to both soluble and mechanical stimuli, such as substrate stiffness and cytokines [11, 38, 39, 40, 41, 42, 43, 44, 45]. In mouse models, TRPV4 deficiency has been linked to altered pressure responses, osteogenesis, fibrosis, and inflammation [28, 34, 35, 36, 40, 41, 42, 43, 44, 45]. Moreover, TRPV4 dysregulation is implicated in human diseases such as skeletal dysplasia and sensory and motor neuropathies [46]. While limited studies have explored TRPV4's role in aortic tissue, some evidence points to elevated TRPV4 protein levels in diseased aortic valves compared to healthy ones, though the underlying mechanisms remain unclear [47, 48]. Additionally, increased TRPV4 expression in the myocardium of diabetic mice is linked to cardiac fibrosis, indicating a broader role for TRPV4 in CVDs that may be mediated through ECM regulation in aECs [11].

In this context, our findings provide novel insights into TRPV4's role in aECs. Specifically, we demonstrate that: (i) TRPV4 is functional in mouse aECs; (ii) genetic ablation or pharmacological inhibition of TRPV4 blocks intracellular matrix stiffening, traction force generation, and EndMT induced by matrix stiffness and TGFβ1; (iii) the N‐terminal residues 100–130 of TRPV4 are essential for intracellular matrix stiffening, cellular traction force generation, MLC2 phosphorylation, and EndMT; and (iv) TRPV4 regulates matrix stiffness‐induced MLC2 activity, thereby modulating EndMT and cellular force generation. These results reveal a critical mechanotransduction role for TRPV4 in regulating EndMT and suggest that TRPV4 represents a promising therapeutic target for addressing CVDs.

## 2 Materials and Methods

### 2.1 Reagents and Antibodies

For Western blot and immunofluorescence microscopy analyses, the following reagents and antibodies were used: Phospho‐MLC2 (p‐MLC2; 3674S), p‐Smad2, Smad2, p‐Smad3, Smad3, p‐AKT, AKT, YAP/TAZ, and MLC2 (3672S) antibodies were obtained from Cell Signaling Technologies (Danvers, MA), while the anti‐alpha smooth muscle actin (α‐SMA) antibody (A2547‐2ML) was purchased from Sigma (St. Louis, MO). Anti‐TRPV4 antibody (ACC‐034) and its corresponding blocking peptide (BLPCC‐034) were sourced from Alomone Labs. Whole‐cell lysates were prepared using Pierce RIPA buffer (89900) and Halt protease and phosphatase inhibitor cocktail (78442), both from Thermo Scientific. For fluorescent detection, Alexa Fluor 488‐conjugated secondary antibodies (A11001) and Alexa Fluor Phalloidin 594 (A12381) were used, along with ProLong Gold Diamond mounting media with DAPI (#P36962) from Invitrogen. TRPV4 antagonist GSK2193874 (GSK219, cat# SML0942), TRPV4 agonist GSK1016790A (GSK101, cat# 6433), and MLC2 antagonist ML‐7 were purchased from Sigma. TGFβ1 (#240‐B) was sourced from R&D Systems. Cell passaging and adhesion removal utilized Gibco's 0.25% Trypsin–EDTA solution (#25‐300‐120). Cells were cultured in Gibco's Dulbecco's Modified Eagle Medium (DMEM/F‐12, 11320‐033) supplemented with heat‐inactivated fetal bovine serum (FBS, 10082‐147) and antibiotic/antimycotic solution from Sigma (A5955). Culture‐grade bovine serum albumin (BSA, SH30574.02) was obtained from HyClone. For traction force microscopy (TFM) studies, Softwell TFM plates with embedded fluorescent beads (SW24G‐COL‐25‐STO.2R‐ER) were used. Polyacrylamide (PA) hydrogels of defined stiffness (1 and 50 kPa) coated with collagen type I (SS24‐COL‐1‐EA, SS24‐COL‐50‐EA) were procured from Matrigen for stiffness‐related experiments. Collagen type I (cat# 804622‐20ML) and collagenase II solution (cat# C2‐22‐1G) were purchased from Sigma. Adenoviral vectors for empty controls, full‐length mouse TRPV4 (Adeno‐RGD‐mouseTRPV4 WT), and three deletion constructs (Ad‐TRPV4‐del‐1‐30, Ad‐TRPV4‐del‐1‐130, and Ad‐TRPV4‐del‐100‐130) were prepared by Vector Biolabs. For calcium influx studies, the FLIPR Calcium 6 assay kit was obtained from Molecular Devices.

### 2.2 Animals

TRPV4 knockout (KO) mice on a C57BL/6 background, originally generated by Dr. Makoto Suzuki (Jichi Medical University, Tochigi, Japan), were obtained from Dr. David X. Zhang (Medical College of Wisconsin, Milwaukee, WI). Congenic wild‐type (WT) mice were procured from Charles River Laboratories. All mice were housed and bred in a temperature‐ and humidity‐controlled, germ‐free environment with ad libitum access to food and water. All experiments adhered to the guidelines set by the Institutional Animal Care and Use Committee (IACUC), and animal protocols were approved by the University of Maryland College Park review committee.

### 2.3 Aortic Endothelial Cell (aEC) Culture and Maintenance

Wild‐type (WT) and TRPV4 knockout (KO) mice were dissected, and the aorta was isolated from the aortic root to the renal bifurcation. The aorta was then longitudinally opened using microscopic scissors to expose the endothelial lining. Tissue sections were thoroughly washed with sterile PBS and treated with collagenase II for 30 min at 37°C. Following this initial incubation, the tissues were minced using a sterile scalpel and subjected to a second 15‐min incubation in collagenase II at 37°C. After the second incubation, the tissue and cell mixture were pipetted several times to dissociate the cells from the tissue surface. The cells were then washed with sterile PBS and resuspended in complete F‐12 HAM medium containing 20% FBS, antibiotics, antimycotics, and l‐glutamine. The cell suspension was plated on collagen‐coated culture dishes and maintained with fresh F‐12 HAM medium replaced every 48 h. For passaging, 0.25% trypsin–EDTA solution was used, and cells were cryopreserved in a solution of 90% FBS and 10% DMSO in liquid nitrogen for future use.

All experiments were conducted using cells at passages 3–8. Cells were cultured in F‐12 HAM medium supplemented with 20% FBS for adenovirus transfection experiments and TGFβ1 treatments. For TGFβ1 treatments without adenovirus transfection, 1% BSA‐containing DMEM was used. This protocol was adapted with reference to a published methodology in the *Journal of Visualized Experiments* (JOVE) [49].

### 2.4 Atomic Force Microscopy (AFM)

We utilized a JPK Nanowizard 4 Atomic Force Microscope (AFM; Bruker Nano GmbH, Berlin, Germany) to assess the stiffness and topography of aECs. Stiffness measurements of living cells were performed in Quantitative Imaging (QI) mode, an advanced force‐spectroscopy‐based technique that enables simultaneous nanomechanical characterization and sample imaging [28, 34, 35, 50]. For this, aECs were transduced with adenoviral TRPV4 constructs or vector controls for 48 h. The cells were then seeded onto glass‐bottom, poly‐d‐lysine‐coated 35 mm petri dishes (FD35PDL, WPI) and treated with or without 5 ng/mL TGFβ1 for 48 h. For live‐cell stiffness measurements, the cells were maintained in PBS buffer at 37°C with 5% CO_2_ and imaged by acquiring QI maps at a resolution of 128 × 128 pixels. For high‐resolution imaging, cells were fixed with 3% paraformaldehyde following a 24‐h treatment with or without TGFβ1, and QI maps were acquired at a resolution of 512 × 512 pixels. All QI measurements were conducted using qpBioAC‐CI‐CB2 cantilevers (Nanosensors) with the following specifications: a nominal resonance frequency of 50 kHz in air, a spring constant of 0.1 N/m, partial gold coating on the detector side, and a circular symmetric quartz probe with a 30 nm radius. For each experimental condition, 10 cells were imaged, with 3–4 areas scanned per cell at an imaging setpoint of 0.5 nN. The sensitivity and spring constant of each cantilever were calibrated individually before measurements. The Young's modulus was calculated using JPK Data Processing software by fitting the Hertz contact mechanics model with a spherical indenter shape to the acquired force curves [28, 34, 35, 50]. This approach provided detailed insights into the mechanical properties of aECs under different experimental conditions.

### 2.5 Traction Force Microscopy (TFM)

Aortic ECs (aECs) were cultured overnight at 37°C on collagen‐coated (10 μg/mL) 25 kPa polyacrylamide (PA) hydrogels embedded with 0.2 μm fluorescent beads in 24‐well plates. Wild‐type (WT) and TRPV4 knockout (KO) aECs were treated with TGFβ1 (5 ng/mL) for 48 h. For adenoviral experiments, TRPV4 KO aECs were transfected with adenoviral constructs, with or without the TRPV4 antagonist GSK219 or the MLC2 inhibitor ML‐7. Fluorescent images of the beads were captured using a microscope with a 20× objective both before and after cell lysis with 0.6% SDS solution. Bright‐field images of the cells were taken prior to the initial fluorescent bead imaging. Bead displacement and the resulting cellular traction forces were analyzed using TractionForAll software [50].

### 2.6 Intracellular Ca^2+^ Influx Measurement

For the Ca^2+^ influx study, we used the FlexStation 3 instrument to measure GSK1016790A (GSK101)‐induced Ca^2+^ influx, a known TRPV4 agonist, in WT and TRPV4 KO aECs cultured in 96‐well black‐wall clear‐bottom plates [28, 34, 35]. Prior to the experiment, cells were pre‐treated with or without the TRPV4 antagonist GSK219 for 1 h. The FLIPR Calcium 6 Assay Kit was used to quantify changes in intracellular Ca^2+^ levels. aECs were washed and incubated with the Calcium 6 dye solution (prepared in 1× HBSS containing 20 mM HEPES and 2.5 mM probenecid) for 45 min at 37°C. Following incubation, the plates were transferred to the FlexStation 3 instrument to measure Ca^2+^ influx in response to GSK101. A23187 (a calcium ionophore) was used as a positive control.

### 2.7 Western Blot Analysis

Whole‐cell extracts were prepared from WT and TRPV4 KO aECs cultured on collagen‐coated plastic tissue culture plates, with or without TGFβ1 treatment for 48 h, using RIPA buffer supplemented with protease and phosphatase inhibitor cocktails. The cell lysates were resolved on a 12% SDS‐PAGE, transferred onto a PVDF membrane, and probed with primary antibodies. The blots were visualized using horseradish peroxidase‐conjugated anti‐rabbit IgG secondary antibodies (1:2000) and developed using the UVP BioSpectrum imaging system. Quantification of the blot images was performed using UVP data processing software.

### 2.8 Overexpression of Full Length and Mutant TRPV4 Gene in TRPV4 KO aECs by Adenovirus Construct Transfection

For gain‐of‐function studies in TRPV4 KO aECs, cells were transfected with a full‐length WT Ad‐TRPV4 construct and three deletion constructs of varying lengths of the mouse TRPV4 gene (Ad‐TRPV4‐Δ1‐30, Ad‐TRPV4‐Δ1‐130, and Ad‐TRPV4‐Δ100‐130) [28, 34, 35]. TRPV4 KO aECs were seeded onto cell culture plates or traction force microscopy plates in DMEM supplemented with 10% FBS. The cells were transfected with adenoviral TRPV4 constructs or an empty vector control (10^7^ plaque‐forming units/ml) for 24 h, followed by a medium replacement with fresh DMEM. The cells were then incubated for an additional 24–48 h in complete DMEM, with or without TGFβ1 treatment (5 ng/mL).

### 2.9 Immunofluorescence Microscopy Analysis

To analyze the production of α‐SMA, F‐actin, p‐MLC2, CD31, and VE‐CAD, WT and TRPV4 KO aECs were cultured on collagen‐coated glass coverslips with or without TGFβ1 treatment (5 ng/mL). To examine the effect of matrix stiffness on the production of these markers, aECs were cultured on polyacrylamide hydrogels of 1 and 50 kPa stiffness, coated with collagen, with or without TGFβ1 treatment. Cells were fixed with 4% paraformaldehyde and incubated in a blocking solution containing 3% FBS and 0.1% Triton X‐100. Primary antibodies against α‐SMA, p‐MLC2, CD31, and VE‐CAD were used at a 1:100 dilution, followed by secondary antibodies at a 1:200 dilution. F‐actin was labeled using Alexa Fluor 594‐conjugated phalloidin, and nuclei were stained with DAPI‐containing mounting medium. To confirm the specificity of the TRPV4 antibody in aECs, WT aECs were cultured on glass coverslips, fixed with 4% paraformaldehyde, and stained with a TRPV4 primary antibody (1:200) and Alexa Fluor‐conjugated anti‐rabbit secondary IgG (1:300), with or without the addition of a TRPV4 blocking peptide.

## 3 Results

### 3.1 TRPV4 Expressed in Mouse Primary aECs Is Functional and Can Be Modulated by Treatment With Its Agonists and Antagonists

**FIGURE 1:**
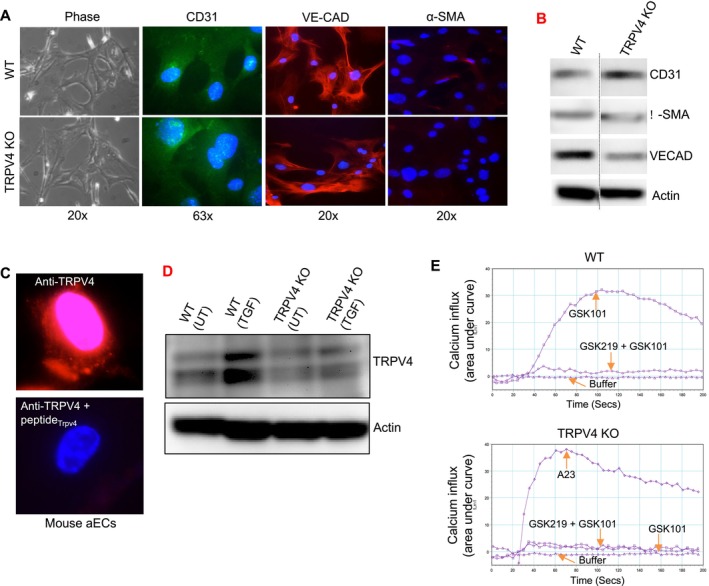
TRPV4 expressed in aortic endothelial cells is functional and can be regulated by its agonist and antagonist treatment. (A) WT and TRPV4 KO normal mouse primary aECs were cultured on collagen‐coated (10 μg/mL) plastic tissue culture plates and were fixed and incubated with anti‐CD31, VE‐CAD, α‐SMA antibodies. Phase and immunofluorescent microscopy and F‐Actin staining by Alexa fluor phalloidin 594 were performed to visualize the expression of endothelial (CD31, VE‐CAD) and mesenchymal markers (α‐SMA and F‐Actin). (B) Immunoblot analysis to confirm the results in Figure A. (C) WT aECs were cultured on plastic plates and were fixed and incubated with anti‐TRPV4 antibody with or without TRPV4 blocking peptide. Immunofluorescent microscopy was performed to visualize the expression of TRPV4 in the aECs. (D) Immunoblot analysis to confirm the TRPV4 loss in TRPV4 KO cells in the presence and absence of TGFβ1 (5 ng/mL, 48 h) treatment. (E) WT and TRPV4 KO aECs (20 000 cells/well) were seeded on collagen‐coated (10 μg/mL) 96‐well plastic plates. TRPV4 agonist GSK101‐induced intracellular Ca^2+^ influx is shown by relative fluorescence units measuring the area under the curve. A23187 (A23; 2 μM), a calcium ionophore, was used as a positive control. FlexStation 3 recording of Calcium 6 dye‐loaded WT and TRPV4 KO aEC monolayers showing the effect of TRPV4 agonist GSK101 and antagonist GSK219 on Ca^2+^ influx.

To confirm that the cultured cells were endothelial cells, we performed immunofluorescence microscopy using endothelial markers CD31 and VE‐CAD, as well as the mesenchymal marker α‐SMA. As expected, both WT and TRPV4 KO cells strongly expressed CD31 and VE‐CAD, while α‐SMA expression was negligible, indicating the absence of mesenchymal characteristics (Figure 1A). Immunoblot analysis of CD31, α‐SMA, and VECAD was performed to confirm the above‐stated immunofluorescence results (Figure 1B). Next, the expression of the TRPV4 channel in aECs was validated using a TRPV4‐specific antibody performing immunofluorescence microscopy (Figure 1C) and immunoblot analysis (Figure 1D). To assess the functionality of TRPV4 channels in aECs, we conducted a calcium influx assay using the Calcium 6 kit and FlexStation 3 to measure intracellular calcium levels. HBSS buffer without calcium served as a negative control, and the A23187 (A23) ionophore was used as a positive control for calcium influx detection. Upon treatment with GSK101 (a TRPV4 agonist), WT cells exhibited a significant increase in calcium influx. However, pretreatment with GSK219 (a TRPV4 antagonist) for 1 h completely inhibited calcium influx in WT cells (Figure 1E). In TRPV4 KO cells, calcium influx was impaired even in the presence of GSK101 (Figure 1E). These findings confirm that TRPV4 channels are functional in aECs and can be effectively modulated by treatment with agonists and antagonists.

### 3.2 TRPV4 Is Essential for Matrix Stiffness‐Mediated EndMT

**FIGURE 2:**
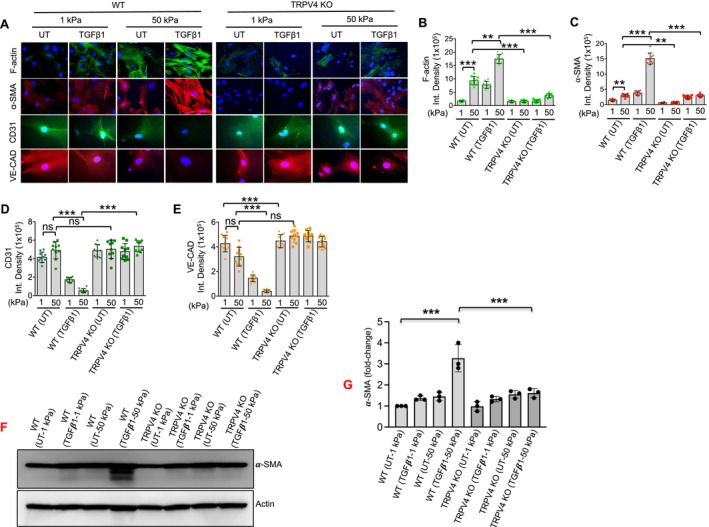
TRPV4 Is essential for matrix stiffness‐mediated EndMT. (A) WT and TRPV4 KO aECs were plated on collagen‐coated soft (1 kPa) and stiff (50 kPa) polyacrylamide hydrogels and incubated with or without TGFβ1 (5 ng/mL, 48 h). Representative images from five different fields per condition showing morphological changes of ɑ‐SMA (red) and F‐Actin (green), VE‐CAD (red) and CD31 (green) with DAPI (blue) staining in WT and TRPV4 KO aECs in response to increasing matrix stiffness and TGFβ1 treatment. (B–E) represents bar diagram representing the quantification of the fluorescent intensity of F‐Actin, α‐SMA, CD31 and VE‐CAD respectively in WT and TRPV4 KO aECs cultured on soft and stiff hydrogel metrices with or without TGFβ1 treatment (results from Figure A). Data are expressed as mean ± SEM, *n* = 20 cells/condition; UT = untreated; **p < 0.01; ***p < 0.001, 1‐way ANOVA followed by Bonferroni test. (F) Immunoblot analysis to confirm the immunofluorescence data shown in panel A. (G) Bar graphs show quantification of results from F. Data analyzed using One‐way ANOVA followed by Bonferroni's multiple comparison test; ***p < 0.001.

During EndMT, cells lose their endothelial markers and acquire mesenchymal markers [1, 2, 3, 4, 5]. Previous studies have highlighted the critical role of matrix stiffness in regulating TRPV4 activity and driving morphological transitions in various cell types, including fibroblasts, dermal epithelial cells, and macrophages [12, 28, 29, 30, 31, 32, 34, 35, 36, 37]. To explore whether matrix stiffness influences EndMT by modulating the expression of endothelial markers, we cultured WT and TRPV4 KO aECs on soft (1 kPa) and stiff (50 kPa) PA hydrogel‐coated coverslips, designed to mimic the stiffness of healthy and diseased aortic tissue. The cells were treated with or without TGFβ1 for 48 h. We used immunofluorescence microscopy to examine the expression of endothelial markers (CD31 and VE‐CAD) and mesenchymal markers (α‐SMA and F‐actin) in fixed cells. Our findings showed that WT aECs treated with TGFβ1 exhibited significantly increased expression of α‐SMA and F‐actin on the soft matrix, along with decreased expression of VE‐CAD and CD31 on the stiff matrix (Figure 2A–E). Notably, the absence of TRPV4 resulted in reduced production of α‐SMA and F‐actin and sustained expression of VE‐CAD and CD31, regardless of matrix stiffness (Figure 2A–E). Immunoblot analysis of α‐SMA was carried out to confirm the immunofluorescence data (Figure 2F,G). These results suggest that TRPV4 expression and function, in combination with matrix stiffness, play a critical role in regulating EndMT.

### 3.3 Activity of TRPV4 Is Directly Related to EndMT via Its N Terminal 100–130 Amino Acid Sequence

**FIGURE 3:**
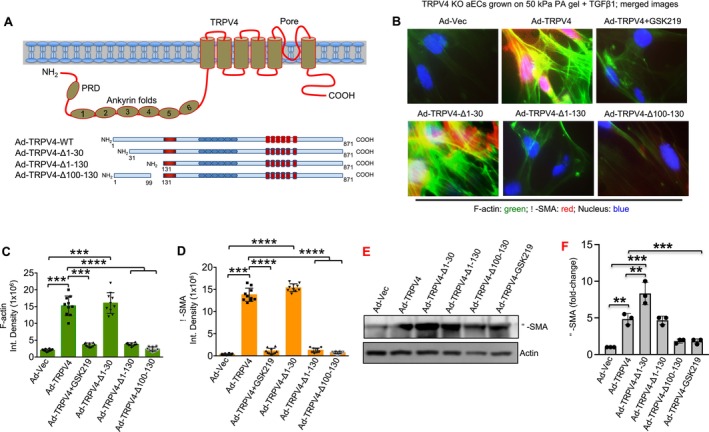
Activity of TRPV4 is directly related to EndMT via its N‐terminal 100–130 amino acid sequence. (A) A schematic diagram depicting the structure of TRPV4 as a transmembrane channel and the design of the deletion constructs (N‐terminal 1–30, 1–130, 100–130) used to overexpress TRPV4 in TRPV4 KO aECs. (B) TRPV4 KO aECs were plated on collagen‐coated stiff (50 kPa) polyacrylamide hydrogels and incubated with TGFβ1 (5 ng/mL, 48 h). Representative images from five different fields per condition showing morphological changes of ɑ‐SMA (red) and F‐Actin (green) and with DAPI (blue) staining of TRPV4 KO aECs in response to overexpression of three deferent adeno constructs of TRPV4 with or without antagonist (GSK219) treatment. (C, D) Represent bar diagrams showing the quantification of the fluorescent intensity of F‐Actin, and α‐SMA respectively in TRPV4 KO aECs cultured on stiff hydrogel matrices with TGFβ1 treatment with the overexpression of full‐length TRPV4 with or without the antagonist and with the expression of three deletion constructs of TRPV4. Data are expressed as mean ± SEM, *n* = 20 cells/condition; ***p < 0.001, ****p < 0.0001, 1‐way ANOVA followed by Bonferroni test. (E) Immunoblot analysis to confirm the immunofluorescence data shown in panel B. (F) Bar graphs show quantification of results from E. Data analyzed using One‐way ANOVA followed by Bonferroni's multiple comparison test; **p < 0.01, ***p < 0.001.

Our initial investigation demonstrated that TRPV4 plays a critical role in TGFβ‐mediated EndMT. To further explore this, we investigated whether restoring TRPV4 function in TRPV4 KO aECs could induce the expression of mesenchymal markers, similar to WT aECs. TRPV4 KO aECs were cultured on collagen‐coated 50 kPa PA hydrogel coverslips and treated with TGFβ1 for 48 h, alongside the following transfections: Ad‐Vec (control), Ad‐TRPV4, three TRPV4 deletion constructs (Ad‐TRPV4‐Δ1‐30, Ad‐TRPV4‐Δ1‐130, and Ad‐TRPV4‐Δ100‐130), and Ad‐TRPV4 plus GSK219 (TRPV4 antagonist) (Figure 3A). These specific deletion constructs were selected based on prior studies in other cell types, which highlighted the functional importance of these domains in TRPV4 activation in response to biomechanical stimuli [34, 42]. Immunofluorescence microscopy analysis revealed that overexpression of TRPV4 in TRPV4 KO cells significantly increased the production of mesenchymal markers α‐SMA and F‐actin compared to control cells (Figure 3B–D). Interestingly, the expression of mesenchymal markers was markedly reduced in cells transfected with TRPV4 deletion constructs lacking the 1–130 or 100–130 amino acid sequences (Figure 3B–D). However, deletion of the 1–30 amino acid sequence did not impair the production of α‐SMA and F‐actin (Figure 3B–D). We performed immunoblot analysis of α‐SMA to confirm the immunofluorescence data (Figure 3E,F). These findings suggest that TRPV4 is a key regulator of the mesenchymal transition in aECs. TRPV4 activity can be restored in TRPV4 KO cells through TRPV4 overexpression, and the N‐terminal 30–100 amino acid region is identified as a critical functional domain mediating TRPV4's role in EndMT. Collectively, these results underscore a direct role for TRPV4 in driving EndMT.

### 3.4 TRPV4 Regulates Intracellular Stiffness Generation in aECs

**FIGURE 4:**
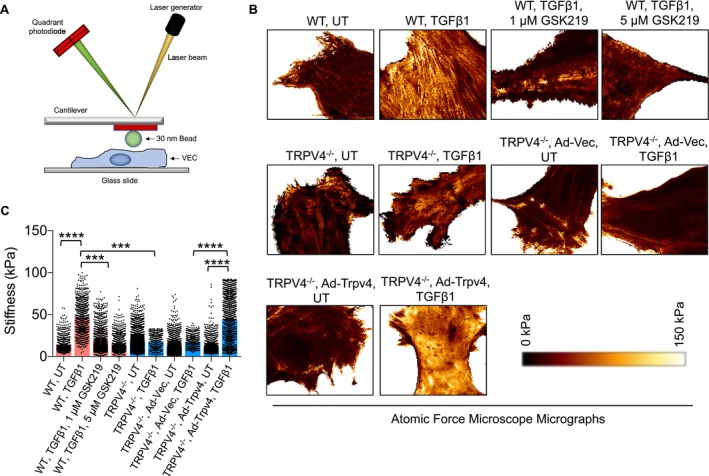
TRPV4 regulates intracellular stiffness generation in aECs. (A) A schematic diagram shows atomic force microscopy (AFM) arrangements to determine the Young's modulus (stiffness) of aECs. A laser beam deflected by the deformation of a cantilever attached to a 30 nm circular symmetric quartz probe was captured by a detector. Young's modulus values (500/condition) were acquired by fitting individual force curves to the Hertz model. (B) AFM micrographs show heat maps representing a variation of stiffness inside and near lamellipodia–filopodial areas of indicated cell groups. Scale bars: 5 μm. (C) Quantification of Young's modulus of the dataset. One‐way ANOVA followed by Bonferroni test; ***p < 0.001, ****p < 0.0001.

Cells generate force to perform essential functions such as migration, communication with neighboring cells, ECM remodeling, and dynamic shape changes [16, 17, 18, 19, 23, 25, 26, 27, 30, 31, 34, 35, 37, 51]. Previous studies have shown that the production of α‐SMA and F‐actin is closely linked to intracellular force generation [34, 51, 52], prompting us to investigate this further using atomic force microscopy (AFM) analysis (Figure 4A). To evaluate the role of TRPV4 in intracellular stiffness generation, we cultured WT and TRPV4 KO aECs with or without TGFβ1 treatment for 24 h. Statistical analysis of AFM data revealed that TRPV4 KO aECs exhibited significantly lower stiffness in response to TGFβ1 treatment compared to WT aECs under the same conditions (Figure 4B,C). Next, we tested whether inhibiting TRPV4 with its antagonist, GSK219, could attenuate stiffness generation. WT aECs were treated with TGFβ1 and either vehicle (control) or GSK219 at two concentrations (1 and 5 μM). We observed that GSK219‐treated cells displayed significantly reduced stiffness compared to vehicle‐treated cells (Figure 4B,C). To further confirm that TRPV4 directly mediates stiffness generation in response to TGFβ1, we employed a gain‐of‐function approach [28, 34]. TRPV4 KO aECs were transfected with an adenoviral vector expressing TRPV4 (Ad‐TRPV4), with an empty adenoviral vector serving as a negative control. Following TGFβ1 treatment, we found that TRPV4 overexpression successfully restored stiffness generation in TRPV4 KO cells (Figure 4B,C). Together, these findings demonstrate that TRPV4 activity is essential for intracellular stiffness generation in aECs and that its role can be modulated through either inhibition or overexpression.

### 3.5 TRPV4 Is Directly Involved in Traction Force Generation in aECs

**FIGURE 5:**
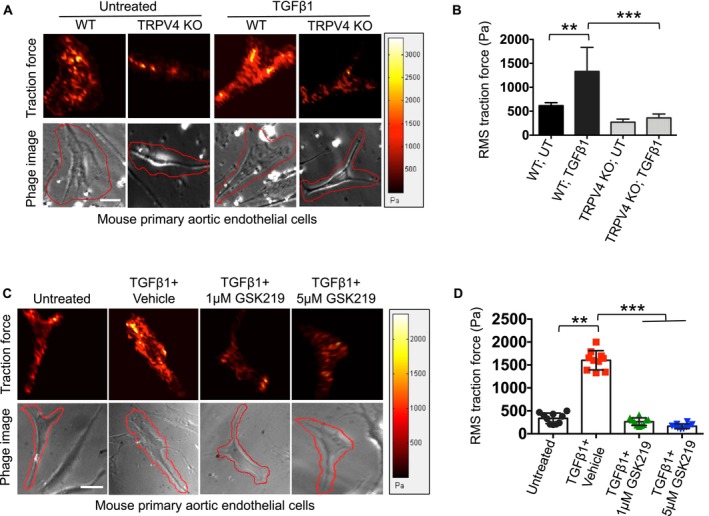
TRPV4 regulates traction force generation in aECs. (A) WT and TRPV4 KO aECs were plated on collagen‐coated (10 μg/mL) 25 kPa PA hydrogels containing 0.2 μm fluorescent beads in 24‐well plates with or without 5 ng/mL of TGFβ1 for 24 h. Color‐coded traction vector maps indicate the magnitude of the traction vector, and corresponding phase images show the cells. (B) Quantitation of results from TFM assay as shown in Figure A. Data are expressed as mean ± SEM, *n* = 10 cells/condition; **p < 0.01, ***p < 0.001, Student's *t*‐test; RMS: Root mean square. (C) WT aECs were plated on collagen‐coated 25 kPa PA hydrogels containing 0.2 μm fluorescent beads in 24‐well plates with or without 5 ng/mL of TGFβ1 for 24 h and with or without two doses of TRPV4 antagonist GSK219 (1 and 5 μM). Color‐coded traction vector maps indicate the magnitude of the traction vector and corresponding phase images show the cells. (D) Quantitation of results from TFM assay as shown in Figure C. Data are expressed as mean ± SEM, *n* = 10 cells/condition; **p < 0.01, ***p < 0.001, Student's *t*‐test; RMS, root mean square.

To examine the direct role of TRPV4 in traction force generation, we cultured WT and TRPV4 KO aECs on 25 kPa hydrogels embedded with fluorescent beads in 24‐well plates. Cells were treated with or without TGFβ1 for 48 h. Statistical analysis revealed that TRPV4 KO aECs generated significantly lower traction forces compared to WT aECs under both untreated and TGFβ1‐treated conditions (Figure 5A,B). Next, we tested whether TRPV4 inhibition could modulate traction force generation. WT aECs were treated with TGFβ1 and either vehicle (control) or GSK219 (TRPV4 antagonist) at two concentrations (1 and 5 μM). Interestingly, we observed that GSK219‐treated cells exhibited significantly reduced traction forces compared to vehicle‐treated cells (Figure 5C,D). These results support the hypothesis that TRPV4 contributes to traction force generation in aECs.

**FIGURE 6:**
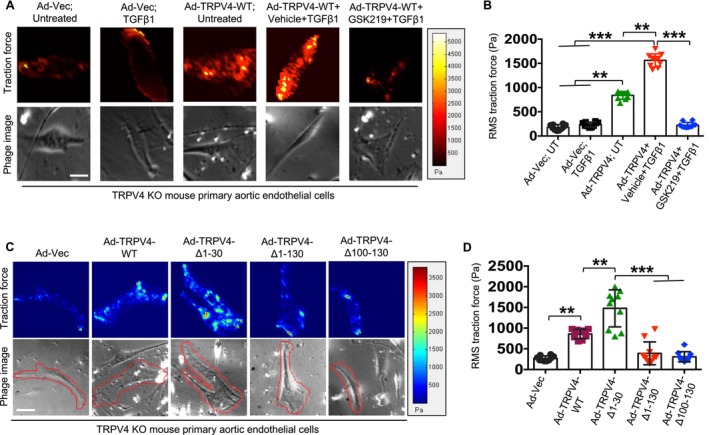
TRPV4 is directly related to traction force generation in aECs, and N‐terminal 100–130 amino acid residues are required for traction force generation. (A) TRPV4 KO aECs were plated on collagen‐coated (10 μg/mL) 25 kPa PA hydrogels containing 0.2 μm fluorescent beads in 24‐well plates with or without 5 ng/mL of TGFβ1 for 48 h. Cells were transfected with full‐length adeno‐TRPV4 or vector control, with or without GSK219 treatment (1 μM). Color‐coded traction vector maps indicate the magnitude of the traction vector, and corresponding phase images show the cells. (B) Quantitation of results from the TFM assay as shown in Figure A. Data are expressed as mean ± SEM, *n* = 10 cells/condition; **p < 0.01, ***p < 0.001, Student's *t*‐test; RMS: Root mean square. (C) TRPV4 KO aECs were plated on collagen‐coated 25 kPa PA hydrogels containing 0.2 μm fluorescent beads in 24‐well plates with 5 ng/mL of TGFβ1 for 48 h. Cells were transfected with full‐length adeno‐TRPV4, 3 adeno mutant constructs of TRPV4, or vector control. Color‐coded traction vector maps indicate the magnitude of the traction vector, and corresponding phase images show the cells. (D) Quantitation of results from the TFM assay as shown in Figure C. Data are expressed as mean ± SEM, *n* = 10 cells/condition; **p < 0.01, ***p < 0.001, Student's *t*‐test; RMS, root mean square.

To further confirm TRPV4's role in traction force generation, we investigated whether reintroducing TRPV4 into TRPV4 KO cells could restore this function [28, 34]. TRPV4 KO aECs were cultured on 25 kPa fluorescent bead‐coated hydrogels and transfected with either Ad‐TRPV4 or Ad‐Vec (control) with or without TGFβ1 treatment. To determine if TRPV4‐mediated traction force could be reversed, we treated cells transfected with Ad‐TRPV4 and TGFβ1 with GSK219. Consistent with our hypothesis, Ad‐TRPV4 transfection significantly increased traction force in TRPV4 KO cells compared to Ad‐Vec‐transfected controls (Figure 6A,B). This effect was further enhanced by TGFβ1 treatment. However, treatment with GSK219 significantly reduced traction force in Ad‐TRPV4‐transfected cells, even in the presence of TGFβ1 (Figure 6A,B). Together, these findings demonstrate that TRPV4 expression is directly linked to traction force generation in aECs, and its activity can be modulated by TRPV4 antagonists.

### 3.6 N‐Terminal 100–130 Amino Acid Residues of TRPV4 Are Required for Traction Force Generation in aECs2

The N‐terminal cytosolic residues of TRPV4 are known to play a critical role in the activation and function of this ion channel [34, 42]. To identify the specific domain responsible for traction force generation, we transfected TRPV4 KO aECs with a full‐length TRPV4 gene, three deletion constructs, and a vector control. The culture conditions for traction force microscopy were maintained as previously described, with cells allowed to grow for 48 h post‐transfection before analysis. Our results revealed that cells transfected with the 1–130 and 100–130 TRPV4 deletion constructs exhibited significantly lower traction force generation compared to those transfected with the full‐length TRPV4 gene (Figure 6C,D). In contrast, deletion of the 1–30 amino acid residues did not affect traction force generation (Figure 6C,D). These findings strongly suggest that the N‐terminal 100–130 amino acid region of TRPV4 is critical for its functionality in traction force generation and its potential role in EndMT, a mechanosensitive process.

### 3.7 Phospho‐MLC2 Is Involved in TRPV4‐Mediated EndMT

**FIGURE 7:**
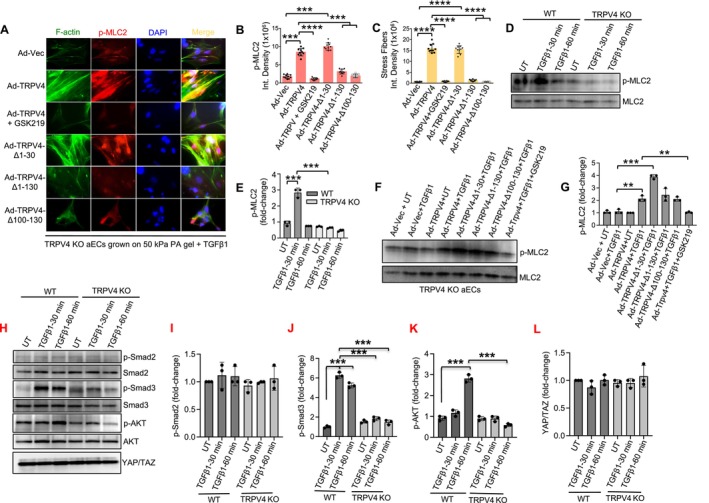
Phospho‐MLC2 is involved in TRPV4‐mediated EndMT and N‐terminal 100–130 amino acid residues of TRPV4 are required for phosphorylation of MLC2. (A) TRPV4 KO aECs were culture on stiff (50 kPa) PA hydrogels. Representative images from five different fields per condition showing morphological changes and co‐localization of F‐Actin (green) and p‐MLC2 (red) with DAPI (blue) staining in TGFβ1 (5 ng/mL)‐stimulated TRPV4 KO aECs transfected with Ad‐TRPV4‐WT (with or without GSK219), Ad‐TRPV4‐Δ1‐30, Ad‐TRPV4‐Δ1‐130, Ad‐TRPV4‐Δ100‐130, or Ad‐Vec. (B, C) Quantitation of results from Figure A. Data are expressed as mean ± SEM, *n* = 20 cells/condition; 1‐way ANOVA followed by Bonferroni test; ***p < 0.001, ****p < 0.0001. (D) WT and TRPV4 KO aEC were treated with TGFβ1 (5 ng/mL) for 30 and 60 min or kept untreated (UT) for the control group. Immunoblotting assay and quantification of p‐MLC2 (Thr18/Ser19) relative to total MLC2 protein levels are shown. All experiments performed three times. (E) Bar graphs show quantification of results from D. Data analyzed using One‐way ANOVA followed by Bonferroni's multiple comparison test; ***p < 0.001. (F) TRPV4 KO aECs were plated on collagen‐coated (10 μg/mL) 50 kPa PA hydrogels and then treated with or without 5 ng/mL of TGFβ1 for 30 min. Cells were transfected with Ad‐TRPV4‐WT (with or without GSK219), Ad‐TRPV4‐Δ1‐30, Ad‐TRPV4‐Δ1‐130, Ad‐TRPV4‐Δ100‐130, or Ad‐Vec. Immunoblotting assay and quantification of p‐MLC2 (Thr18/Ser19) relative to total MLC2 protein levels are shown. All experiments performed three times. (G) Bar graphs show quantification of results from D. Data analyzed using One‐way ANOVA followed by Bonferroni's multiple comparison test; **p < 0.01, ***p < 0.001. (H) WT and TRPV4 KO aECs were treated with TGFβ1 (5 ng/mL) for 30 and 60 min or kept untreated (UT) for the control group. Immunoblotting assay of p‐Smad2, Smad2, p‐Smad3, Smad3, p‐AKT, AKT, and YAP/TAZ are shown. All experiments performed three times. (I–L) Bar graphs show quantification of results from H. Data analyzed using One‐way ANOVA; ***p < 0.001.

Myosin light chain‐2 (MLC2) is a calcium‐binding protein that regulates stretch‐induced contractility and movement in cardiac and vascular cells [53, 54, 55, 56]. Its activation is mediated through phosphorylation in a calcium‐dependent manner [54, 55, 56]. Studies have linked the phosphorylation of this cytoskeleton‐associated protein to cell differentiation [57, 58]. In our study, we sought to investigate the role of phosphorylated MLC2 (p‐MLC2, Thr18/Ser19) in EndMT, focusing on its relationship with F‐actin production. TRPV4 KO aECs were cultured on glass coverslips with or without TGFβ1 treatment for 48 h. The cells were transfected with Ad‐Vec (control), three TRPV4 deletion constructs, or the full‐length TRPV4 gene, with and without GSK219 treatment. Fluorescent imaging of F‐actin, p‐MLC2, and DAPI revealed a significant increase in the co‐localization of F‐actin and p‐MLC2 in Ad‐TRPV4‐transfected cells compared to the vector control (Figure 7A–C). Notably, cells transfected with TRPV4 deletion constructs lacking the 1–130 or 100–130 amino acid regions exhibited substantially reduced levels of F‐actin and p‐MLC2. Consistent with our previous findings [34], the deletion of the 1–30 amino acid region had no significant effect on MLC2 phosphorylation or its co‐localization with F‐actin (Figure 7A–C). Immunoblot analysis of p‐MLC2 in whole‐cell lysates from WT and TRPV4 KO aECs, either untransfected or transfected with Ad‐Vec or TRPV4 constructs, corroborated these observations (Figure 7D–G). Further results show that in WT cells, compared to TRPV4 KO aECs treated with TGFβ1 (5 ng/mL) or left untreated (UT) for the control group, the levels of p‐Smad3 and p‐AKT were upregulated, while the levels of YAP/TAZ and p‐Smad2 remained unaffected (Figure 7H–L). Together, these findings strongly suggest that p‐MLC2, p‐Smad3, and p‐AKT act downstream of TRPV4 in the regulation of EndMT, emphasizing the importance of the 100–130 amino acid region of TRPV4 in this process.

### 3.8 Phospho‐MLC2 Is Involved in TRPV4‐Mediated EndMT and Traction Force Generation in aECs

**FIGURE 8:**
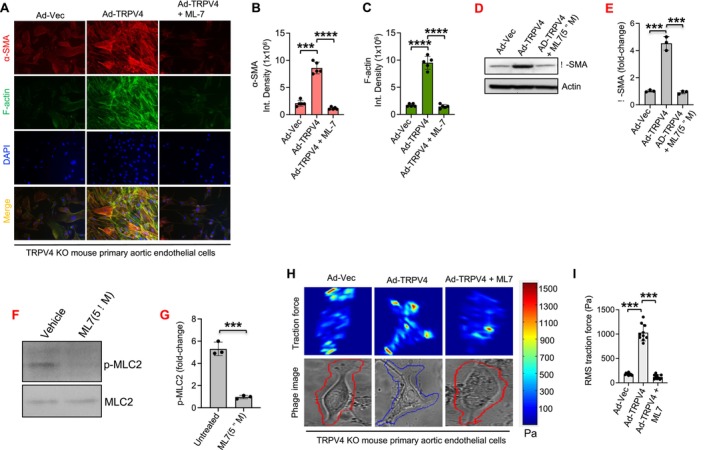
Phospho‐MLC2 is linked to TRPV4‐mediated EndMT and traction force generation in aECs. (A) TRPV4 KO aECs were culture on stiff (50 kPa) PA hydrogels. Representative images from five different fields per condition showing morphological changes and co‐localization of F‐Actin (green) and ɑ‐SMA (red) with DAPI (blue) staining in TGFβ1 (5 ng/mL)‐stimulated TRPV4 KO aECs transfected with Ad‐TRPV4‐WT (with or without MLC2 inhibitor ML‐7) or Ad‐Vec. (B, C). Quantitation of results from Figure A. Data are expressed as mean ± SEM, *n* = 20 cells/condition; 1‐way ANOVA followed by Bonferroni test; ***p < 0.001, ****p < 0.0001. (D). Immunoblot analysis to confirm the ɑ‐SMA immunofluorescence data shown in panel A. (E) Bar graphs show quantification of results from D. Data analyzed using One‐way ANOVA; ***p < 0.001. (F) Immunoblot analysis to confirm that ML‐7 is attenuating p‐MLC2 levels. (G) Bar graphs show quantification of results from F. Data analyzed using *t*‐test; ***p < 0.001. (H) TRPV4 KO aECs were plated on collagen‐coated (10 μg/mL) 25 kPa PA hydrogels containing 0.2 μm fluorescent beads in 24‐well plates with 5 ng/mL of TGFβ1 for 48 h. Cells were transfected with full‐length adeno‐TRPV4 (with or without MLC2 inhibitor ML‐7) or Ad‐Vec. Color‐coded traction vector maps indicate the magnitude of the traction vector, and corresponding phase images show the cells. (I) Quantitation of results from TFM assay as shown in Figure D. *n* = 10 cells/condition; ***p < 0.001, Student's *t*‐test; RMS, root mean square.

In this study, we investigated the role of p‐MLC2 in EndMT. TRPV4 KO aECs were cultured with or without TGFβ1 treatment for 48 h and transfected with Ad‐Vec or the full‐length TRPV4 gene, with or without ML‐7, an inhibitor of p‐MLC2. Immunofluorescence microscopy revealed that TRPV4 overexpression in TRPV4 KO cells significantly increased the production of mesenchymal markers α‐SMA and F‐actin compared to the control (Figure 8A–C). Interestingly, the expression of mesenchymal markers was substantially reduced in TRPV4‐overexpressing KO cells pretreated with ML‐7 (Figure 8A–C). We performed immunoblot analysis to confirm the ɑ‐SMA immunofluorescence data (Figure 8D,E). Immunoblot analysis presented in Figure 8F,G shows that ML‐7 treatment is attenuating p‐MLC2 levels. Furthermore, traction force microscopy showed that TRPV4 overexpression in TRPV4 KO cells significantly enhanced traction force generation compared to Ad‐Vec‐transfected cells. However, pretreatment with ML‐7 notably diminished traction force generation in TRPV4‐overexpressing cells (Figure 8H,I). These findings suggest that TRPV4 regulates MLC2 activity, thereby modulating both EndMT and cellular force generation. This highlights a potential mechanism by which TRPV4 activity regulates p‐MLC2 and force generation to drive EndMT.

## 4 Discussion

The findings of this study underscore the critical role of matrix stiffness and mechanosensitive TRPV4 channels in regulating EndMT, a process with profound implications for cardiovascular health and disease. Our results demonstrate that TRPV4 activity, particularly through its N‐terminal amino acid sequence (100–130), is a key mediator of stiffness‐induced mechanical signaling in aECs. These insights add to the growing body of evidence linking mechanotransduction pathways to the progression of CVDs, particularly atherosclerosis.

Our findings confirm that matrix stiffness acts as a pivotal modulator of EndMT by influencing intracellular mechanical cues such as cytoskeletal tension and traction force generation. These mechanical signals are closely linked to the biochemical activation of key pathways, including TGFβ1 signaling, which is enhanced in the context of a stiffened ECM [23, 24, 25, 26, 27, 28]. The interplay between stiffness‐induced cytoskeletal remodeling, actomyosin contraction, and enhanced phosphorylation of mesenchymal markers like α‐SMA and fibronectin reinforces the hypothesis that mechanical feedback loops amplify EndMT. The involvement of TRPV4 channels as a central stiffness sensor introduces an additional layer of complexity to these regulatory mechanisms.

This study highlights a novel role for TRPV4 channels in mediating stiffness‐induced EndMT and provides mechanistic insights into how these channels regulate cellular stiffness and traction force generation. The specific requirement for the N‐terminal amino acid sequence (100–130) in TRPV4‐mediated responses suggests that this region may serve as a critical determinant for channel activity and its downstream effects. Our findings further reveal that TRPV4 activity modulates the phosphorylation of MLC2, Smad3, and AKT, known regulators of cytoskeletal tension and force generation [53, 54, 55, 56, 57, 58]. This establishes a direct link between TRPV4‐mediated calcium signaling and the mechanical processes driving EndMT.

The discovery that TRPV4 channels regulate intracellular stiffness and traction forces in aECs has significant implications for understanding the mechanistic basis of CVD progression. Matrix stiffening is a hallmark of atherosclerosis and other fibrotic diseases [23, 24, 25, 26, 27, 28, 29, 30, 31, 32, 33, 34, 35, 36, 59, 60, 61], and the role of TRPV4 as a stiffness sensor positions it as a critical mediator of pathological remodeling in these conditions. Targeting TRPV4 activity may provide a novel therapeutic approach to mitigating EndMT and its downstream effects, thereby addressing the fibrotic processes that contribute to vascular dysfunction.

While our study establishes TRPV4 as a key mechanosensor and regulator of EndMT, several questions remain. First, the precise molecular mechanisms by which the N‐terminal amino acid sequence of TRPV4 influences channel activity and mechanotransduction require further investigation. Additionally, exploring how TRPV4 interacts with other mechanosensitive pathways, such as integrin‐mediated adhesion [53], could provide a more comprehensive understanding of EndMT regulation. Finally, in vivo studies are needed to validate the therapeutic potential of TRPV4 inhibition in mitigating vascular stiffening and atherosclerosis.

Our study advances the understanding of how matrix stiffness and TRPV4‐mediated mechanotransduction contribute to EndMT, uncovering critical molecular pathways that link mechanical and biochemical signals in aECs. These findings highlight TRPV4 as a promising therapeutic target for addressing matrix stiffness‐driven pathologies, particularly in the context of cardiovascular disease. By elucidating the mechanistic underpinnings of EndMT, this work provides a foundation for developing novel strategies to combat atherosclerosis and its associated complications.

## Author Contributions

S.O.R. conceived the study, designed and performed the experiments, analyzed data, and written and edited the MS. P.M. wrote the materials and methods section of the manuscript and performed the experiments. S.G.R. analyzed data, wrote and edited the manuscript; K.R.S. performed and analyzed immunoblot experiments; and M.I.K. performed and analyzed the calcium influx experiment. All authors reviewed the manuscript and approved the final content of the manuscript.

## Funding

This work was supported by HHS|NIH|National Institute of Allergy and Infectious Diseases (NIAID) (Grant R01EB024556).

## Conflicts of Interest

The authors declare no conflicts of interest.

## Data Availability

All data generated or analyzed during this study is included in this article.
